# Predicting tuberculosis drug resistance with machine learning-assisted Raman spectroscopy

**Published:** 2023-06-09

**Authors:** Babatunde Ogunlade, Loza F. Tadesse, Hongquan Li, Nhat Vu, Niaz Banaei, Amy K. Barczak, Amr. A. E. Saleh, Manu Prakash, Jennifer A. Dionne

**Affiliations:** 1 Department of Materials Science and Engineering, Stanford University; Stanford, 94305, CA, USA.; 2 Department of Bioengineering, Stanford University School of Medicine and School of Engineering; Stanford, 94305, CA, USA.; 3 Department of Mechanical Engineering, Massachusetts Institute of Technology; Cambridge, 02142, MA, USA.; 4 The Ragon Institute, Massachusetts General Hospital; Cambridge, 02139, MA, USA.; 5 Department of Applied Physics, Stanford University; Stanford, 94305, CA, USA.; 6 Pumpkinseed Technologies, Inc; Palo Alto, 94306, CA, USA.; 7 Department of Pathology, Stanford University School of Medicine; Stanford, 94305, CA, USA.; 8 Division of Infectious Diseases, Massachusetts General Hospital; Boston, 02114, MA, USA.; 9 Department of Medicine, Harvard Medical School; Boston, 02115, MA, USA.; 10 Department of Engineering Mathematics and Physics, Cairo University; Giza, 12613, Egypt.; 11 Department of Radiology, Molecular Imaging Program at Stanford (MIPS), Stanford University School of Medicine; Stanford, 94035, CA, USA.

## Abstract

Tuberculosis (TB) is the world’s deadliest infectious disease, with 1.5 million annual deaths and half a million annual infections. Rapid TB diagnosis and antibiotic susceptibility testing (AST) are critical to improve patient treatment and to reduce the rise of new drug resistance. Here, we develop a rapid, label-free approach to identify *Mycobacterium tuberculosis* (Mtb) strains and antibiotic-resistant mutants. We collect over 20,000 single-cell Raman spectra from isogenic mycobacterial strains each resistant to one of the four mainstay anti-TB drugs (isoniazid, rifampicin, moxifloxacin and amikacin) and train a machine-learning model on these spectra. On dried TB samples, we achieve > 98% classification accuracy of the antibiotic resistance profile, without the need for antibiotic co-incubation; in dried patient sputum, we achieve average classification accuracies of ~ 79%. We also develop a low-cost, portable Raman microscope suitable for field-deployment of this method in TB-endemic regions.

## One Sentence Summary

Raman spectroscopy and machine learning can be used to rapidly identify and differentiate strains of antibiotic-resistant tuberculosis (TB) with high accuracy, on a low-cost platform suitable for deployment in TB-endemic regions.

## INTRODUCTION

The discovery of antibiotics in the early 20^th^ century marked a turning point in our defense against tuberculosis (TB) – one of the deadliest infectious diseases known to humans. At that time, TB had become curable and was considered to be on a path toward elimination. However, by the end of the 20th century, TB re-emerged as a leading cause of death globally, in part due to challenges in diagnosis and its evolved resistance to antibiotics. While growth in liquid culture is considered the gold standard for organism identification and determination of antibiotic susceptibility, the causative organism, *Mycobacterium tuberculosis* (Mtb) can take up to 40 days to culture. As antibiotic susceptibility testing requires pathogen growth, definitive determination of Mtb antibiotic resistance using traditional antibiotic susceptibility testing (AST) can take additional weeks. In the delay between diagnosis and AST results, many patients are treated with only partially active antibiotic regimens, giving rise to resistant strains. In 2021 alone, more than half a million cases of multidrug resistant TB infections were reported (1).

To mitigate the rise of antimicrobial resistance, a key strategy of the World Health Organization (WHO) is to improve surveillance of antibiotic-resistant infections, and to promote the appropriate use of quality medicines (2). To address WHO guidelines, several culture-free AST approaches are being developed. For example, nucleic acid amplification tests and polymerase chain reaction (PCR) do not require culturing and are highly sensitive and specific. However, these tests are limited to identifying resistance in cases in which a defined number of known genomic mutations confer resistance (e.g., the rpoB gene for resistance to rifampicin (3)). These assays also require multiple reagents, can suffer from errors with each thermal cycle, and cannot distinguish between live and dead bacteria, so they cannot be used to monitor treatment efficacy. Further, these assays cost well above the targeted $5000 in capital cost specified by the WHO for next generation TB drug-susceptibility testing tools (4). Complementing PCR, loop-mediated isothermal amplification (LAMP) assays eliminate the need for thermal cycling. However, these LAMP assays cannot detect mutations in resistance-associated genes because of their inability to resolve single-nucleotide differences (5). Rapid lateral flow point-of-care test antigen/antibody detection methods provide simpler and faster alternatives to LAMP and PCR but have poor sensitivity and specificity. There is also a significant delay in the appearance of target antigens in sputum or antibodies in the bloodstream, and these antigens do not necessarily indicate Mtb drug susceptibility. Therefore, current AST methods do not collectively provide the speed, sensitivity, and specificity needed in one system to meet WHO’s goals for drug-resistant TB eradication.

As an up-and-coming approach, Raman spectroscopy has the potential to identify the antibiotic resistance profiles of bacteria at the single-cell level using acquisition times on the order of seconds. Raman spectroscopy utilizes inelastic light scattering to probe the vibrational modes of a sample as a fingerprint. Different bacterial phenotypes are characterized by unique biomolecular compositions, leading to subtle differences in their corresponding Raman spectra. The Raman spectra of biological macromolecules typically reside between 500 and 1900 cm^−1^, with lipids, proteins, and nucleic acids exhibiting fingerprints between 1000–1700 cm^−1^, 1200–1660 cm^−1^, and 600–1100 cm^−1^, respectively (6). As such, these unique Raman spectral signatures can be used for accurate cellular detection, identification, and antibiotic susceptibility testing. Using Raman spectroscopy and machine learning (ML) based spectral analysis, we have previously shown that genetically engineered antibiotic resistant and susceptible *S. aureus* strains can be classified with ~89% accuracy (7). Similarly, we and others have used machine learning-assisted Raman to identify over 31 bacterial species and strains (7,8, 9), including pathogens in liquid solvents (10) and in blood (11, 12); to identify substrains of *E.coli* (13, 14), to identify various respiratory viruses (15), to identify various cellular metabolites and secretomes (16), to assess the effects of antibiotics on resistant and susceptible bacteria (17, 18), and to determine *S. aureus* antibiotic susceptibility without co-incubation (19). Despite these pioneering studies, determination of antibiotic susceptibility of TB, and methods translation to TB-endemic regions, remains an outstanding, untackled challenge.

Here, we demonstrate a rapid, culture-free, and antibiotic co-incubation free antibiotic susceptibility test for TB, based on the integration of Raman spectroscopy and machine learning. Particularly, we classify the antibiotic resistance profile of 5 isogenic Bacillus Calmette-Guérin (BCG) strains using a convolutional neural network. We collect Raman spectra from over 20,000 individual cells resistant to one of the four mainstay anti-TB drugs (isoniazid, rifampicin, moxifloxacin and amikacin) and a pan-susceptible wild type strain. Using only 15-second integration times and Raman features from across the entire spectral range (740–1802 cm^−1^), we achieve >98% resistant versus susceptible classification accuracy across all 5 BCG strains. In addition, we show that various genetic mutants can be accurately classified according to their antibiotic resistance. Using feature recognition algorithms, we also identify the subsets of wavenumbers which most strongly influence antibiotic resistance classification. These algorithms show that antibiotic resistance behavior is primarily reflected in certain vibrational modes – particularly those of mycolic acid - that underlie the molecular-level antibiotic response of the bacteria. These algorithms also show that targeting the entire spectral range of scientific grade Raman instruments is not necessary for TB AST. To this end, we develop a low-cost portable Raman setup that targets select wavenumber bands, for point-of-care applications. Our instrument costs <$5000, and with inclusion of plasmonic nanoantennas for surface-enhanced Raman spectroscopy (SERS), achieves an average accuracy of 89% across all 5 BCG strains. In dried patient sputum samples, our instrument achieves ~79% classification accuracy – providing a foundation for rapid, low-cost, and portable AST testing in resource-limited regions.

## RESULTS

### Single-cell Raman spectroscopy of Mtb for antibiotic incubation-free drug testing

**Figure 1.**
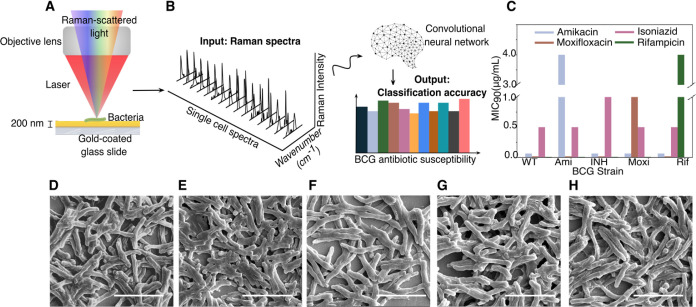
Experimental setup and example bacterial Raman spectra with antibiotic resistance profiles. A) Diffraction-limited spot of a 633 nm incident laser collecting a spectrum from roughly a single bacterial cell. B) Bacterial spectra are used as input for the neural network to perform a classification task that outputs probability scores for each bacterium antibiotic resistance. C) Minimum inhibitory concentrations for the five distinct strains tested across the four main antibiotics using standard serial two-fold dilution. See Table S1 for details. (D, E, F, G) Scanning electron microscopy (SEM) image of a monolayer of wildtype, amikacin-resistant, rifampicin-resistant, isoniazid-resistant, and moxifloxacin-resistant BCG. Scale bar is 4 μm.

As summarized in Figure. 1, we deposit mycobacterial cells onto gold-coated silica substrates and collect single-cell Raman spectra (Fig. 1A). For our studies, we controllably engineer antibiotic-resistant Mtb models using BCG, a live-attenuated form of *Mycobacterium Bovis* as a model organism. While lacking central virulence determinants, BCG is nearly identical to Mtb in its core bacterial functions, including DNA replication, RNA polymerase, and cell wall functions. We use these models to enable safe handling of resistant samples in BSL-2 facilities for the proof-of-concept studies performed here. Individual Raman spectra are used as an input into our convolutional neural network; we employed a 10-fold cross validation scheme to determine the unbiased model performance. The output of this algorithm is a series of probability scores for antibiotic resistance (Fig. 1B). The class with the highest probability score is chosen as the predicted class.

We performed our antibiotic susceptibility test on five isogenic BCG strains: those resistant to the first-line TB antibiotics isoniazid and rifampicin; those resistant to the second-line TB antibiotics moxifloxacin and amikacin; and also on a fully antibiotic-susceptible control strain (hereby described as wildtype). Figure 1C shows the minimum inhibitory concentration of the four anti-TB antibiotics used on our five BCG strains, confirming each resistant strain’s resistance to a single antibiotic as well as the full antibiotic susceptibility of the wildtype strain. As seen in Figures 1D–H, scanning electron micrographs show no distinct morphological difference among the resistant strains.

**Figure 2.**
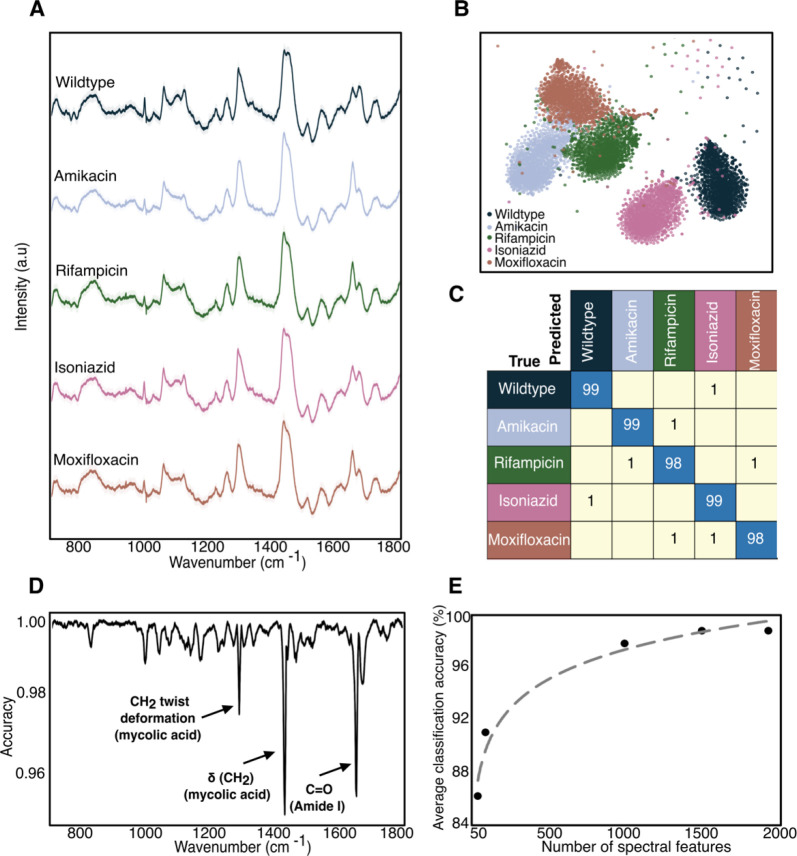
BCG antibiotic resistance determination. A) Average spectrum with standard deviation of all spectrograms (1700 spectra collected at 15s acquisition) from the 5 BCG strains. The spectra show significant differences in both peak position and peak intensity at bands centered at ~1000 cm^−1^, 1060 cm^−1^, and between ~1300–1700 cm^−1^. B) Two-dimensional t-SNE projection across all Raman spectra of the dataset for 5 BCG samples susceptible and resistant to the four different antibiotics tested grouped according to antibiotic class showing clustering. C) Normalized confusion matrix generated using CNN on the single-cell spectra collected from 5 BCG strains, grouped by antibiotic class. Samples were evaluated by performing a stratified K-fold cross validation of our classifier’s performance across 10 splits, showing ~98% classification accuracy across all samples. D) Feature selection highlighting the identification of true, physical vibrational modes. Feature selection performed to determine relative weight of spectral wavenumbers in our CNN classification. E) Average classification accuracy as a function of the number of top spectral features selected showing 96% accuracy with only 500 features.

As shown in Fig. 2A, the average Raman spectra of the 5 distinct BCG strains (~ 1700 spectra per strain) look similar, with major spectral peaks preserved across the strains. However, there are some noticeable differences in the ~1000, ~1300, ~1500 and ~1700 cm^−1^ bands between wildtype and four resistant strains; the majority of these vibrational modes correspond to cell-wall components such as mycolic acid and proteins. Therefore, the differences between the spectra are most likely due to differences in cell-wall composition and structure, discussed later in the text. We performed t-distributed stochastic neighbor embedding (t-SNE) which shows significant clustering for each of the 5 strains (Fig. 2B). Such clustering suggests that accurate classification by resistance should be possible. To classify the antibiotic resistance profiles of the 5 BCG strains, we developed a deep learning model, a ResNet (20) variant (7). We perform a stratified K-fold cross validation of our convolutional neural network’s performance across 10 splits. As seen in Figure 2C, we achieve an average accuracy of 98% across all BCG strains. Note that main diagonal elements represent correct classifications and off-diagonal elements represent misclassifications. High classification accuracies were also obtained with different gold-coated glass slide substrates (Fig. S3). These results demonstrate that our approach is agnostic to the particular substrate selected or batch effects and are solely from intrinsic differences in the strains themselves. Therefore, Raman spectroscopy in conjunction with machine learning can be used to predict antibiotic resistance in mycobacteria without the need for antibiotic co-incubation and hence without culturing.

### Select spectral bands are most crucial for predicting antibiotic resistance

We quantitatively identify the regions of the spectra that are key for the high classification accuracy. To identify these meaningful bands, we used a probing algorithm that perturbs the input data before re-running the classification through the trained models from Fig. 2C (21). Using each test fold from our 10-fold cross validation, we iterate through the wavenumbers and at each iteration, perturb the spectrum by modulating the amplitude of the spectral intensity with a Voigt distribution centered at the probing wavenumber. After each perturbation, we recalculate the classification accuracy, compare the updated results with our baseline classification accuracy, and determine the importance for each wavenumber - the greater the decrease in accuracy due to a given perturbation, the more important the wavenumber. As shown in Fig. 2D, these regions are spectral bands located around 1295, 1437 and 1660 cm^−1^ (22). These wavenumber bands correspond to mycolic acid -CH_2_ twist and -CH_2_ deformation modes, and Amide I C=O stretching modes, which change when the bacteria develop resistance. It is known that single point mutations in relevant genes confer resistance to a given anti-TB drug, such as the GyrA or GyrB genes in moxifloxacin-resistant Mtb as well as the katG gene in isoniazid-resistant Mtb. Therefore, our analysis suggests that the biochemical origins of resistance can be tracked by examining important features in the Raman spectra.

Next, we reduced the wavenumber feature input to our neural network, reflecting those bands most important for high-accuracy classification. Using only the top 50 wavenumbers, we achieve ~86% accuracy. Classification accuracy increases to 96% when using 250 features, and further to 98% when using all 1930 features (Fig. 2E). This result indicates potential for use of a cost-effective spectrometer, compared to the scientific grade tool, targeting these select bands; we use this insight to design our low-cost Raman microscope, discussed later in the text.

### Antibiotic-resistant mutants can be accurately classified

**Figure 3.**
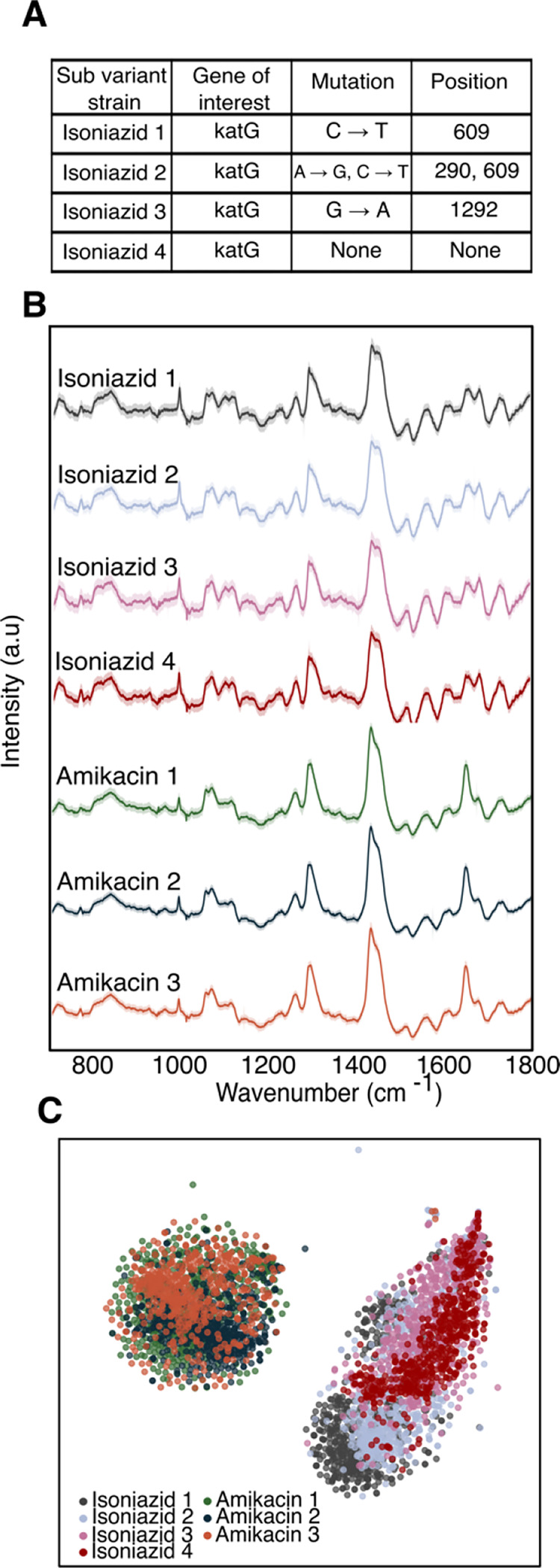
Resistant mutant prediction. A) Sequencing results showing single mutation differences resulting in mutants of resistant strains against isoniazid and amikacin B) Average Raman spectra of 529 single cell spectra with standard deviation of four isoniazid resistant mutants and three amikacin-resistant mutants showing nearly identical fingerprints among their respective class of resistance. C) t-SNE plot showing overlapping clusters of mutants within a class of antibiotic resistance.

To test the robustness of our approach to genetic mutations, we interrogated four isoniazid-resistant mutants and three amikacin-resistant mutants. As seen in Figure 3A, the isoniazid-resistant mutants differ from each other by single point mutations in the katG gene, which encodes for catalase peroxidase, an enzyme that converts isoniazid to its biologically active form (23). Isoniazid resistant mutant 1 has a single point mutation at position 609 from cytosine to thymine; isoniazid resistant mutant 2 has two single point mutations at positions 290 and 609 from adenine to guanine and cytosine to thymine, respectively; isoniazid resistant mutant 3 has a single point mutation at position 1292 from guanine to adenine; and isoniazid resistant mutant 4 has no single point mutations identified in katG, and likely harbors a non-katG mutation conferring the observed isoniazid resistance. As seen in Figure 3B, Raman spectra from these distinct isoniazid-resistant mutants are seemingly identical, with tightly overlapping projections on t-SNE clustering (Fig. 3C). Yet, there is slight separability within the mutant clusters, suggesting that Raman spectroscopy could have the remarkable ability to predict point mutations in the genetic sequence of pathogens, and possibly other cells.

The three amikacin-resistant mutants reveal similar clustering on the t-SNE projection. Importantly, however, the clustering is distinct from the isoniazid cluster. The classification accuracy to predict the antibiotic susceptibility of each strain within its antibiotic class is ~99% (Fig. S2). Clinically, this result is significant, as it is likely that antibiotic-resistant mutants from different patients will differ on the single nucleotide scale, and we are able to identify resistance to a particular antibiotic regardless of this difference.

### Enabling low-cost and portable Raman for Mtb drug testing

**Figure 4.**
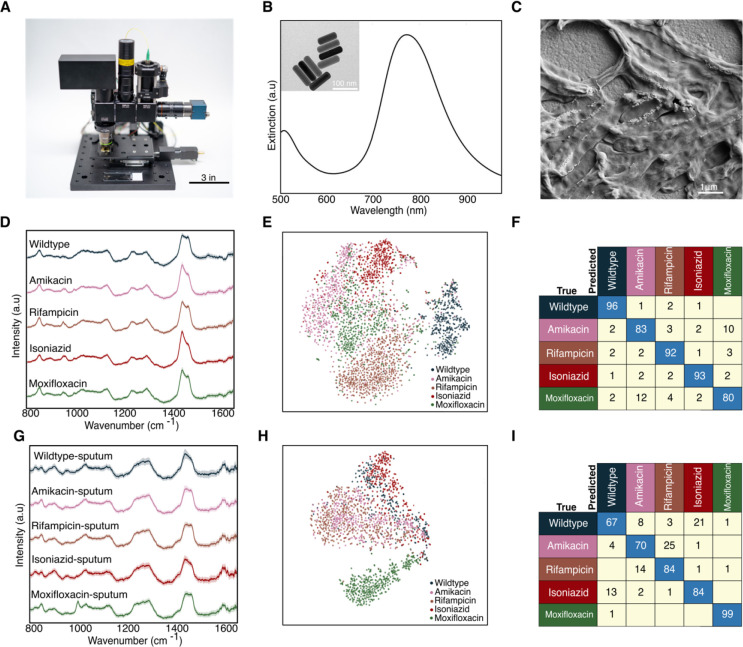
Demonstration on our low-cost Raman system. A) Photo of our system showcasing its small footprint and portable platform for use at the point-of-care. A standard microscope slide is included for scale. B) Extinction spectra of gold nanorods showing plasmon resonance centered at ~780 nm to overlap with the setup’s 785 nm excitation laser. Panel (B) inset shows transmission electron micrograph of gold nanorods used. C) Scanning electron micrograph of BCG mixed with gold nanorods, which increase the Raman scattering from single cells. D) Raman spectra of the 5 BCG strains mixed with gold nanorods imaged on our setup. E) t-SNE projection across all Raman spectra of the 5 BCG strains. F) Average accuracy of ~89% is achieved using spectra from bacteria suspended in water and dropcast dried (replication of Fig. 2c on our setup) G) Raman spectra of the 5 BCG strains spiked in sputum imaged on our setup. H) t-SNE projection across all spiked sputum samples. I) Average accuracy of ~79% is achieved using spectra collected from two separate spiked sputum samples

To demonstrate that our findings can translate to point-of-care TB drug testing in resource-limited regions, we developed a low-cost, portable, and fully automated Raman microscope (Fig. 4A). This Raman microscope is based on the Octopi/Squid modular microscope framework developed by our team for fluorescence (24, 25, 26), but advances this setup for spectroscopy. The instrument utilizes a non-cooled CMOS camera (Sony IMX290), a transmission diffraction grating with >95% efficiency, and a design without a pinhole or slit for low-cost spectroscopy. A fiber-coupled 785 nm VHG-stabilized laser is used as the excitation source. To boost the signal from single bacteria, we synthesize gold nanoparticles (AuNPs) for surface-enhanced Raman scattering. As seen in Figure 4B, these nanorods have a scattering resonance near the pump-laser wavelength. Compared to whole-cell Raman, these nanorods yield a ~2 order of magnitude increase in Raman scattering (9, 10, 11, 27, 28, 29, 30, 31) from single cells and pathogens and considerably shorten the integration times required for single-cell analysis on our microscope. The AuNPs are also relatively inexpensive to synthesize or purchase, easy to tune optically (32, 33, 34) and easy to integrate with our bacterial strains. All components combined for this microscope and spectrometer are <$5000. Additional details on the sample preparation and imaging/spectroscopy are provided in SI.

As a baseline measurement, we first collected ~3000 spectra from the 5 BCG strains mixed with gold nanorods using 0.3 second integration times and 3 accumulations per sample (Fig. 4B). Here, the shortened integration time was chosen to maintain a similar signal-to-noise ratio of the signal as with the Raman of the scientific-grade instrument. Similar to the spectra collected on the scientific grade Raman microscope in Fig. 2A, the average Raman spectra of the 5 distinct BCG strains look similar, with major spectral peaks preserved across the strains. However, there are some noticeable differences in the ~1000, ~1300 and ~1400 cm^−1^ bands between wildtype and four resistant strains (Fig. 4D). We performed t-SNE which shows significant clustering for each of the 5 strains (Fig. 4E). As seen in Figure 4f, we achieve an average accuracy of 89% across all 5 BCG strains, comparable with the ~95–98% average classification accuracy achieved on our scientific-grade tool.

As an initial evaluation of the performance for clinical samples, we spiked BCG pathogens into sputum (see Methods), mixed the sample with gold nanorods, and collected a total of ~5300 spectra from the BCG strains. Using only 0.3 second integration times and 3 accumulations per sample, we obtain an average classification accuracy of ~79% for the 5 strains across two separate sputum samples (Fig. 4I and Fig. S7). This specificity is comparable with that observed for bacteria alone on scientific grade Raman instruments (Fig. 2C) while providing rapid results at a lower cost and smaller footprint.

We note that the spectra differ from those without using AuNPs, which is as expected as now the Raman signatures primarily originate from the surface-bound molecules on the bacteria, due to the electric field localization of the AuNPs. This intraclass spectral variation is evident in the milder, less intense clustering of the 5 strains in the t-SNE in Figure 4E and Figure 4H compared to the whole-cell Raman collected in Figure 2B. However, our convolution neural network can still classify strains, as demonstrated by the high accuracy we are able to obtain. Future work spanning more patient data and optimized spectral data processing could enable clinical BCG strain classification.

## DISCUSSION

In summary, we demonstrate a rapid and accurate TB AST methodology that is antibiotic co-incubation free and culture-free. Unlike current culture-based and nucleic acid amplification-based testing (NAATs) platforms, our Raman approach does not require lengthy culturing, expensive reagents, or thermal cycling equipment, and is robust to single nucleotide point mutations. Our approach can accurately classify resistance across a variety of antibiotics, and across a variety of genetic mutations. In addition, our machine learning models not only provide accurate AST classification, but also interpretability to the Raman spectra. Indeed, as highlighted by our feature-recognition algorithms, resistance behavior is primarily reflected in certain vibrational modes, particularly those of mycolic acid, that underlie the molecular-level antibiotic response of the bacteria. By focusing on the wavenumbers most important for Mtb AST, we developed a low-cost portable Raman microscope, capable of high Mtb classification accuracy on dried samples and in dried sputum. On-going work is aimed at testing our approach in the field on Mtb-positive patients. Such clinical work will elucidate the intrinsic differences between strain types, well beyond sputum-to-sputum variations. If successful, this clinical work will provide a new and needed approach to enable the TB surveillance and diagnostic milestones set by the WHO.

## Supplementary Material

Supplement 1

## Data Availability

All data needed to evaluate the conclusions in the paper are present in the paper and/or the Supplementary Materials. Additional data related to this paper may be requested from the authors.
