# Supplementary material for: Rapid, antibiotic incubation-free determination of tuberculosis drug resistance using machine learning and Raman spectroscopy
Source: ArXiv. 2024 Apr 9:arXiv:2306.05653v2. Originally published 2023 Jun 9. Preprint. [Version 2] (PMC10274949)
Supplement: 1 [file NIHPP2306.05653V2-supplement-1.pdf]

# Supporting Information for Rapid, antibiotic incubation-free determination of tuberculosis drug resistance using machine learning and Raman spectroscopy

**Authors:** Babatunde Ogunlade[1*†], Loza F. Tadesse[2,3,4*], Hongquan Li[5*], Nhat Vu[6], Niaz Banaei[7], Amy K. Barczak[4,8,9], Amr. A. E. Saleh[1,10], Manu Prakash[2] and Jennifer A. Dionne[1,11†]

**Affiliations:**
[1] Department of Materials Science and Engineering, Stanford University; Stanford, 94305, CA, USA.

[2] Department of Bioengineering, Stanford University School of Medicine and School of Engineering; Stanford, 94305, CA, USA.

[3] Department of Mechanical Engineering, Massachusetts Institute of Technology; Cambridge, 02142, MA, USA.

[4] The Ragon Institute, Massachusetts General Hospital; Cambridge, 02139, MA, USA.

[5] Department of Applied Physics, Stanford University; Stanford, 94305, CA, USA.

[6] Pumpkinseed Technologies, Inc; Palo Alto, 94306, CA, USA.

[7] Department of Pathology, Stanford University School of Medicine; Stanford, 94305, CA, USA.

[8] Division of Infectious Diseases, Massachusetts General Hospital; Boston, 02114, MA, USA.

[9] Department of Medicine, Harvard Medical School; Boston, 02115, MA, USA.

[10] Department of Engineering Mathematics and Physics, Cairo University; Giza, 12613, Egypt.

[11] Department of Radiology, Molecular Imaging Program at Stanford (MIPS), Stanford University School of Medicine; Stanford, 94035, CA, USA.

*Indicates equal contribution
† To whom correspondence should be addressed;
E-mail: bogun@stanford.edu; jdionne@stanford.edu.

**This PDF file includes:**

Figures S1 to S13
Table S1

SI References

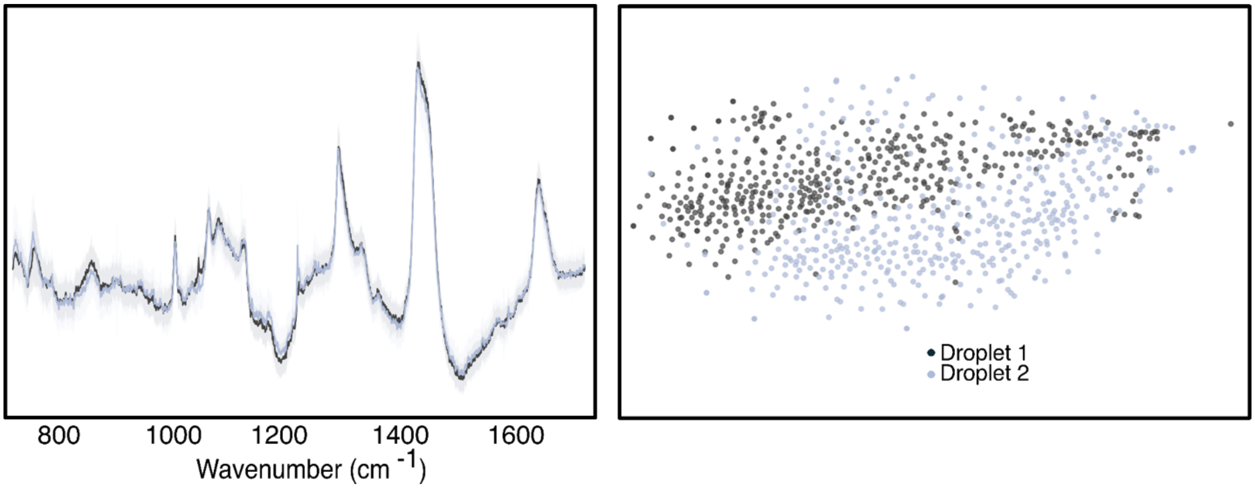


**Fig. S1.** Droplet repeatability study. 450 Raman spectra of wildtype BCG collected on two separate droplets, showing spectral overlap and T-SNE overlap.

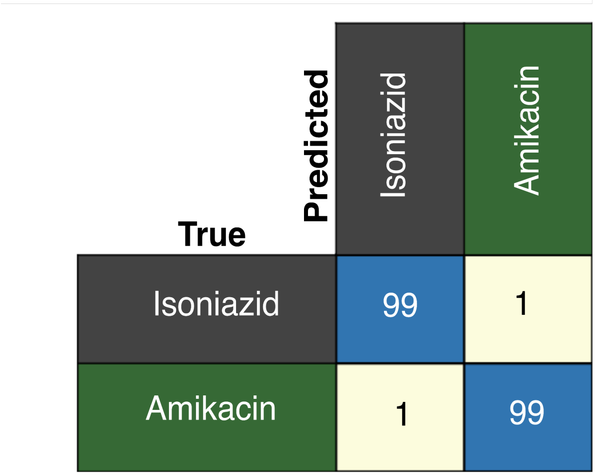


**Fig. S2.** Confusion matrix classifying the isoniazid and amikacin resistant mutants when grouped according to their antibiotic class, showing ~99% classification accuracy (529 spectra per strain; 2116 spectra for the Isoniazid grouping and 1587 spectra for the Amikacin grouping)

| True \ Predicted | Wildtype | Amikacin | Rifampicin | Isoniazid | Moxifloxacin |
|---|---|---|---|---|---|
| Wildtype | 95 | | | 5 | |
| Amikacin | | 97 | 1 | | 2 |
| Rifampicin | 1 | 1 | 95 | 1 | 2 |
| Isoniazid | 5 | | | 94 | 1 |
| Moxifloxacin | | 3 | 2 | | 95 |

| True \ Predicted | Wildtype | Amikacin | Rifampicin | Isoniazid | Moxifloxacin |
|---|---|---|---|---|---|
| Wildtype | 96 | | | 4 | |
| Amikacin | | 98 | | | 2 |
| Rifampicin | 1 | 1 | 95 | 1 | 2 |
| Isoniazid | 5 | | | 95 | |
| Moxifloxacin | | 4 | 2 | 1 | 93 |

| | Wildtype | Amikacin | Rifampicin | Isoniazid | Moxifloxacin |
|---|---|---|---|---|---|
| Wildtype | 98 | | | 1 | 1 |
| Amikacin | | 97 | | 1 | 2 |
| Rifampicin | | | 99 | 1 | |
| Isoniazid | | | 1 | 97 | 2 |
| Moxifloxacin | | 2 | | 4 | 94 |

| | Wildtype | Amikacin | Rifampicin | Isoniazid | Moxifloxacin |
|---|---|---|---|---|---|
| Wildtype | 96 | | 1 | 1 | 2 |
| Amikacin | | 93 | 1 | 5 | 1 |
| Rifampicin | 1 | | 96 | 2 | 1 |
| Isoniazid | 1 | 9 | 2 | 82 | 6 |
| Moxifloxacin | 3 | 3 | 1 | 7 | 86 |

**Fig. S3.** Confusion matrices from four replicate studies on the Horiba Labram scientific grade Raman microscope showing an average of ~95% classification accuracy across the 5 BCG strains ( ~450, 529, ~420, and ~420 spectra per strain collected, respectively).

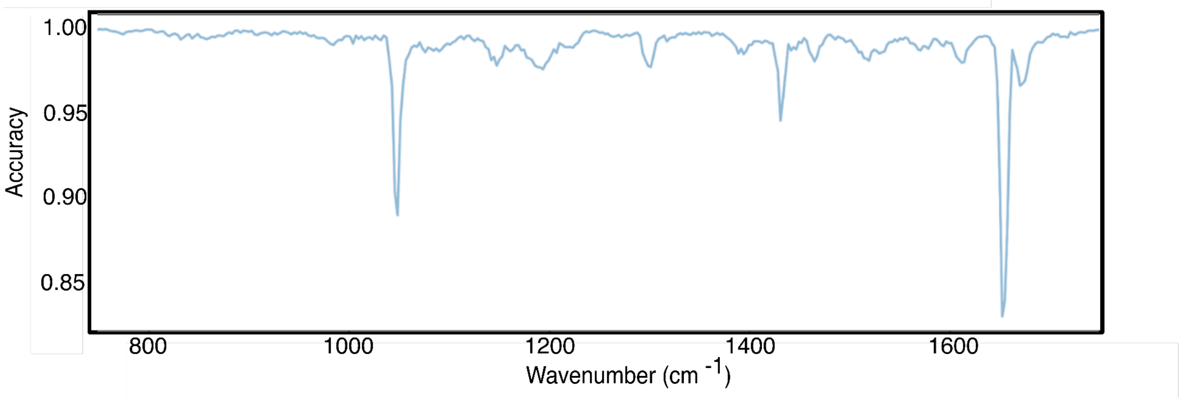

1.00
0.95
0.90
0.85
Accuracy
800
1000
1200
1400
1600
Wavenumber (cm -1)


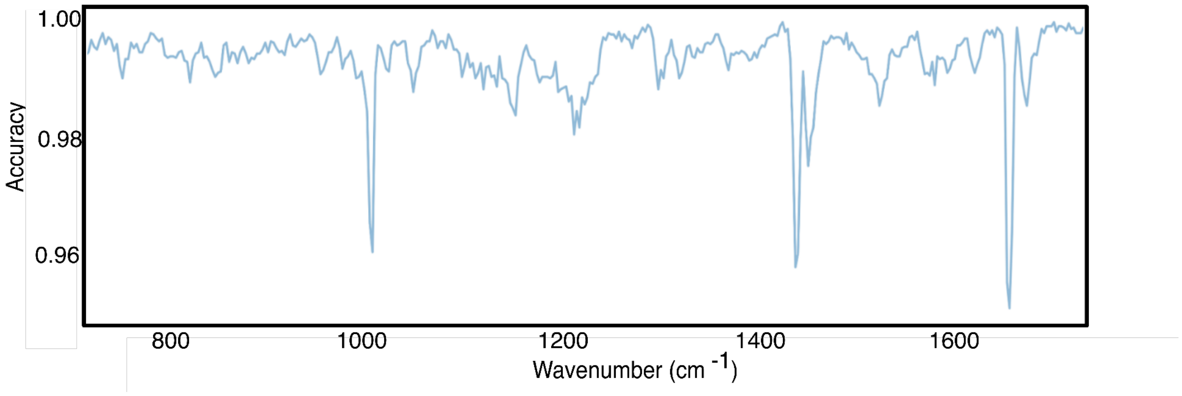

1.00
0.98
0.96
Accuracy
800
1000
1200
1400
1600
Wavenumber (cm -1)


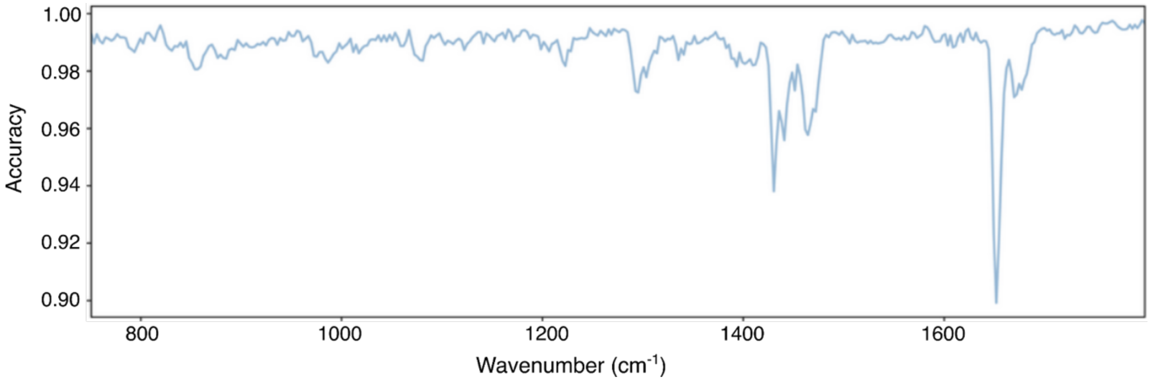

1.00
0.98
0.96
0.94
0.92
0.90
Accuracy
800
1000
1200
1400
1600
Wavenumber (cm-1)

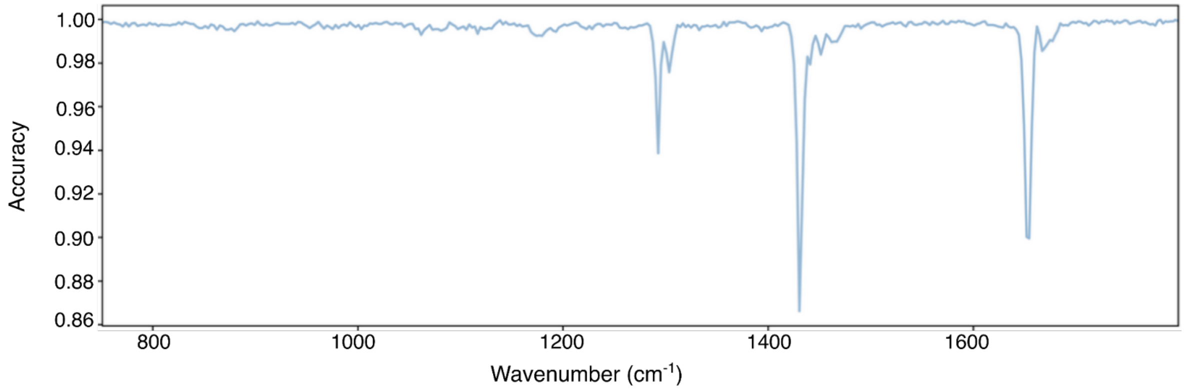


**Fig. S4.** Feature selection from four replicate studies on the Labram.

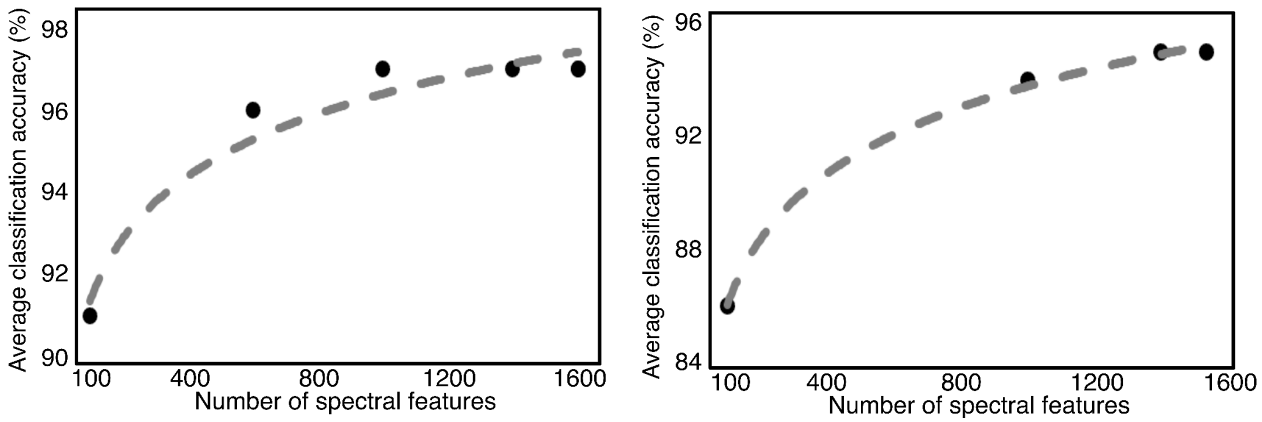


**Fig. S5.** Average classification accuracy vs number of spectral features for two replicate Labram studies.

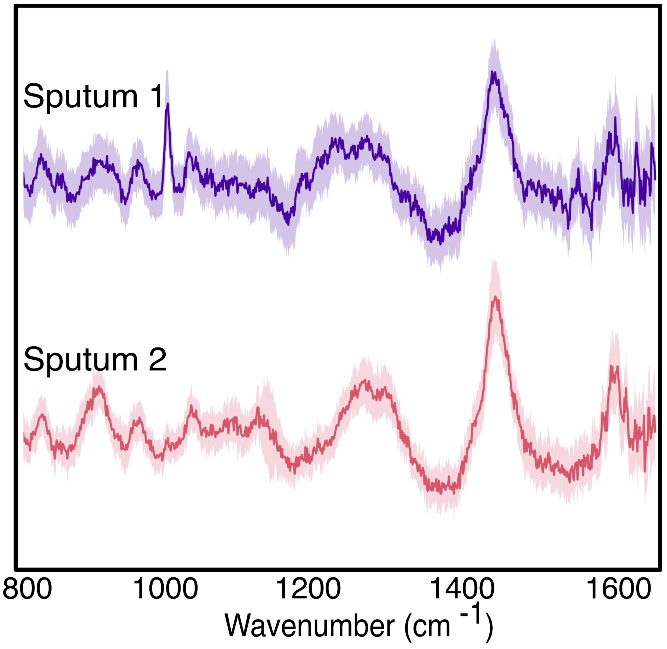

**Fig. S6.** Average Raman spectra of the two sputum samples used in the Octopi study.

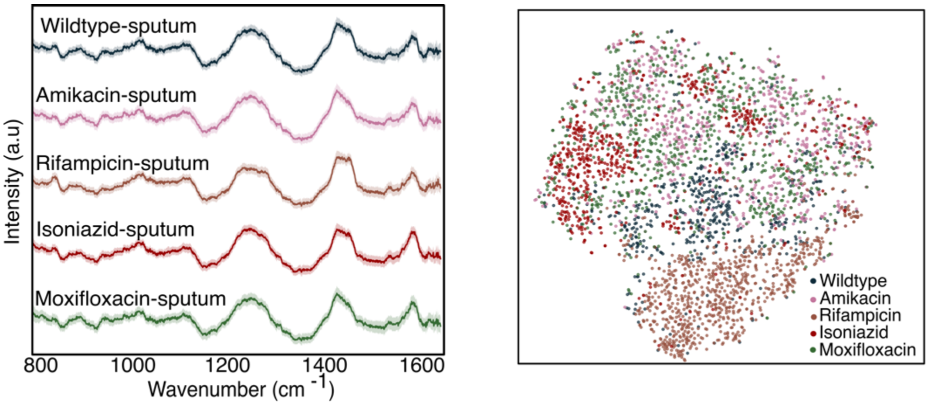


| | Wildtype | Amikacin | Rifampicin | Isoniazid | Moxifloxacin |
|---|---|---|---|---|---|
| Wildtype | 75 | 7 | 6 | 4 | 8 |
| Amikacin | 4 | 63 | 1 | 1 | 31 |
| Rifampicin | 2 | | 96 | 2 | |
| Isoniazid | 5 | 3 | 3 | 83 | 6 |
| Moxifloxacin | 4 | 21 | 2 | 3 | 70 |

**Fig. S7**. Mean Raman spectra of 5 BCG strains spiked in other sputum sample collected on Octopi-Raman with t-SNE showing some clustering by antibiotic class, and the confusion matrix demonstrating ~ 77% average classification accuracy.

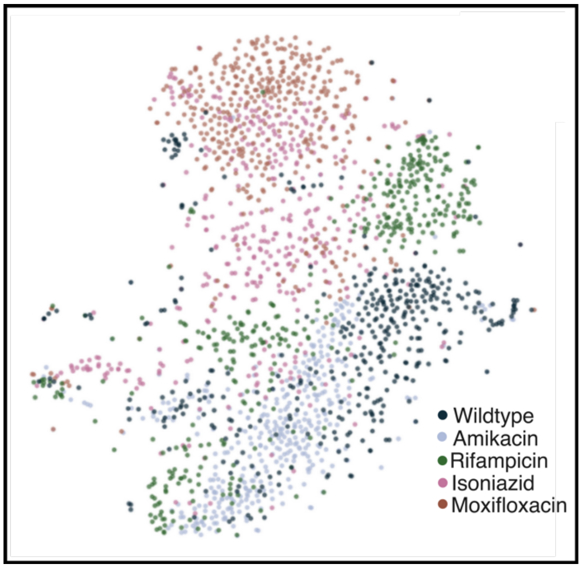


| | Wildtype | Amikacin | Rifampicin | Isoniazid | Moxifloxacin |
|---|---|---|---|---|---|
| Wildtype | 87 | 5 | 1 | 4 | 3 |
| Amikacin | 4 | 92 | 3 | | 1 |
| Rifampicin | 2 | 2 | 90 | 2 | 4 |
| Isoniazid | 2 | 1 | 1 | 86 | 10 |
| Moxifloxacin | 2 | 1 | 5 | 14 | 78 |

**Fig. S8** T-SNE of 5 BCG strains spiked in Sputum 2 collected on the Labram showing clear clustering by antibiotic class, and the confusion matrix demonstrating ~87% average classification accuracy.

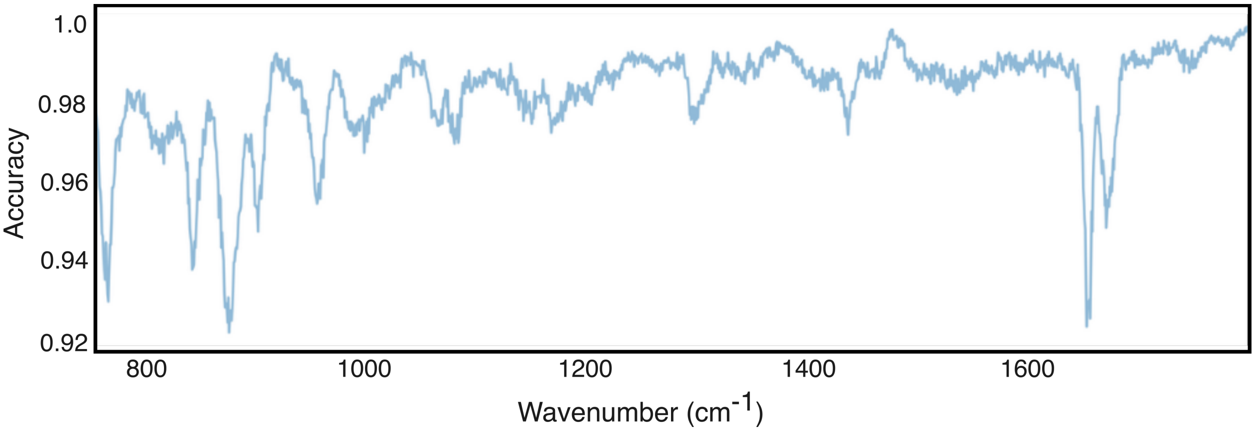


**Fig. S9** Feature selection of 5 BCG strains spiked in Sputum 2 collected on the Labram

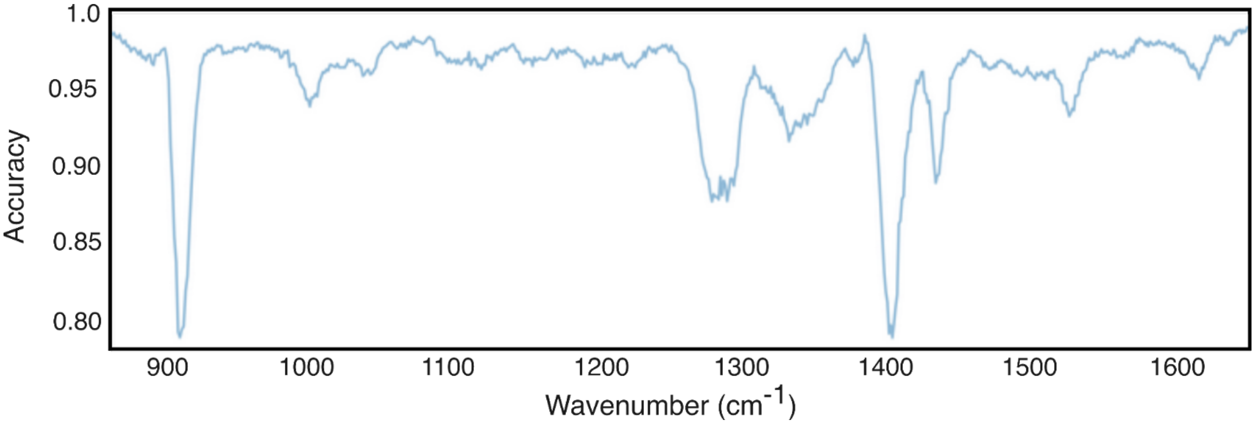


**Fig. S10** Feature selection of 5 BCG strains collected on Octopi-Raman

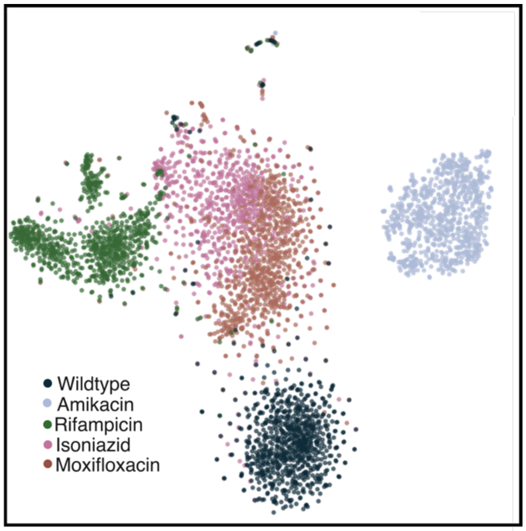


| | Wildtype | Amikacin | Rifampicin | Isoniazid | Moxifloxacin |
|---|---|---|---|---|---|
| Wildtype | 97 | | | 1 | 2 |
| Amikacin | | 99 | | 1 | |
| Rifampicin | 1 | | 95 | 2 | 2 |
| Isoniazid | 1 | | 2 | 87 | 10 |
| Moxifloxacin | 1 | | 2 | 10 | 87 |

**Fig. S11** T-SNE and confusion matrix (~840 spectra/ class) of combined datasets from 5 BCG strains from two experimental replicates

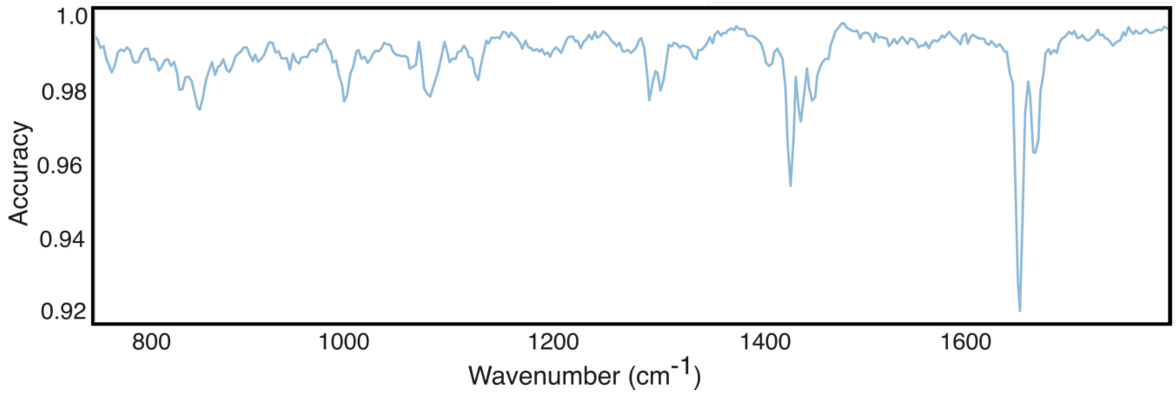


**Fig. S12** Feature selection of combined datasets from 5 BCG strains from two experimental replicates

| BCG strain | Gene of interest | Mutation | Position |
|---|---|---|---|
| Isoniazid 1 | katG | C-->T | 609 |
| Isoniazid 2 | katG | A-->G, C-->T | 290, 609 |
| Isoniazid 3 | katG | G-->A | 1292 |
| Isoniazid 4 | katG | none | none |
| Moxifloxacin 1 | GyrB | C-->T, C-->T | 1167, 1207 |
| Amikacin 1 | rrs | A-->G | 1401 |
| Amikacin 2 | rrs | A-->G | 1401 |
| Amikacin 3 | rrs | A-->G | 1401 |
| Rifampicin | rpoB | T-->C, C-->T | 1325, 1349 |

**Fig S13** List of SNPs present in antibiotic-resistance associated genes of interest for each strain[42]

**Table S1:**

| | $\mathbf{MIC_{90}}(\mu g/mL)$ | | | | |
|---|---|---|---|---|---|
| Antibiotic | Wildtype BCG | Amikacin-resistant BCG | Isoniazid-resistant BCG | Moxifloxacin-resistant BCG | Rifampicin-resistant BCG |
| Amikacin | 0.0625 | $> 4.0$ | 0.0625 | 0.0625 | 0.0625 |
| Isoniazid | 0.5 | 0.5 | $<1$ | 0.5 | 0.5 |
| Moxifloxacin | 0.016 | 0.016 | 0.016 | 1 | 0.016 |
| Rifampicin | $<0.008$ | $<0.008$ | $<0.008$ | $<0.008$ | $<4.0$ |

**Table S1:** Minimum inhibitory concentrations of the five main BCG strains studied. This was obtained using standard serial two-fold dilution method.

**References (40, 41, 42):**


40. X. Ye, C. Zheng, J. Chen, Y. Gao, C. B. Murray, Using binary surfactant mixtures to simultaneously improve the dimensional tunability and monodispersity in the seeded growth of gold nanorods. *Nano Lett.* **13**, 765–771 (2013).

41. K. C. Carroll, M. A. Pfaller, M. L. Landry, A. J. McAdam, R. Patel, S. S. Richter, D. W. Warnock, *Manual of Clinical Microbiology, 2 Volume Set* (Wiley, 2019).

42. National Center for Biotechnology Information (NCBI)[Internet]. Bethesda (MD): National Library of Medicine (US), National Center for Biotechnology Information; [1988] – [cited 2024 March 05]. Available from: https://www.ncbi.nlm.nih.gov/
