# Supplementary material for: Predicting tuberculosis drug resistance with machine learning-assisted Raman spectroscopy
Source: ArXiv. 2023 Jun 9:arXiv:2306.05653v1. Preprint. [Version 1] (PMC10274949)
Supplement: Supplementary file 1 [file NIHPP2306.05653v1-supplement-1.pdf]

Supplementary Information: Predicting tuberculosis drug resistance with machine learning-assisted Raman spectroscopy

Materials and Methods:

BACTERIAL STRAINS

To raise antibiotic resistant mutants, BCG was plated on 7H10 media with increasing concentrations of each antibiotic of interest. Plates were incubated at 37 °C for 21 days. Individual colonies were selected and grown in 7H9 media containing OADC, 0.2% glycerol, and 0.05% Tween-80; antibiotic resistance was confirmed by Minimum inhibitory concentration (MIC) testing.

MIC DETERMINATION

Each strain was grown to mid-log phase in 7H9 media containing OADC, 0.2% glycerol, and 0.05% Tween-80. Cultures were then diluted back to an OD600 of 0.01 in media containing 2-fold dilutions of each antibiotic of interest. Cultures were grown at 37°C in 96-well plates; bacterial cells were mixed at 7 and 14 days of incubation, and growth was determined by OD600 measurements on a Tecan Spark plate reader.

BACTERIAL PREPARATION FOR SPECTROSCOPY

To obtain the antibiotic-resistant BCG strains, wildtype BCG (ATCC 35737 TMC 1019 [BCG Japanese]) was grown to mid-log phase in 7H9 liquid media supplemented with OADC, glycerol, and tween-80, then pelleted and plated on 7H10 solid media containing serial dilutions of each antibiotic of interest from 10-fold above the MIC to 10-fold below the MIC. Plates were incubated at 37 ^{o}C for 4 weeks; individual colonies were then selected for subculture. Particularly, each colony harbors a unique resistance gene and hence was used as a sub-strain for the specific resistance pattern that we studied. To determine MIC, wild-type BCG and each resistant strain was grown to mid-log phase, then diluted back to an OD600 of 0.01 and incubated in two-fold serial dilutions of each antibiotic in a 96-well plate. Days 7 and 14 post-inoculation, each well was gently mixed and OD600 was read on a TECAN Spark device. Only confirmed resistant mutants, as well as the fully-susceptible (wildtype) strains were used for Raman testing. 1 mL of frozen stock of the wildtype and the confirmed resistant mutants were seeded in 10 mL 7H9 liquid media supplemented with OADC, glycerol, and tween-80 and incubated at 37 ^{o}C shaking at 115 RPM until mid-log phase (~5-6 days) using the Thermo Scientific MaxQ 4450 incubator. Cultures were then chemically inactivated overnight in a 1:1 solution using 10% formalin. 1.5 mL of culture (~4 $*10^{8}$ cells/mL) was washed with water three times at 6000 rpm for 4 min using a mySPINTM 6 Mini Centrifuge. The BCG strains were prepared for Raman interrogation by resuspending the final washed pellet in 100 μL of sterile water and drying 3 μL of the suspension on a gold-coated silica substrate. Droplets were dried for 20 minutes and Raman was collected immediately after drying.

For the sputum spiked BCG Raman interrogation, 25 μL of 3x washed bacteria was spun down and resuspended in 50 μL of sputum. After sitting for 1 minute, the spiked sputum was then spun down, washed once with DI water, and then resuspended in 25 μL of DI water. 5 μL of cell solution was mixed with nanorods in a 1:1 ratio, and 3 μL of the suspension was dried on a gold-coated silica substrate.

RAMAN SPECTRAL DATA COLLECTION AND PROCESSING

The BCG strains were illuminated with a 633 nm laser powered at 13.17 mW. The Raman spectra were collected from 742 cm^{-1} to 1802 cm^{-1} using a 100x, 0.9 NA objective with a spot size of ~ 1.5 μm , a 600 l/mm grating, and 15 second acquisition time per spectra, ensuring that spectra are collected from single to at most a few bacterial cells at a time. Python (Jupyter Notebook) was used to process spectral data. Individual spectra were smoothed using wavelet denoising, specifically using the denoise wavelet function from the scikitimage Python library. Background was then subtracted using a polynomial fit with degree 5, and spectra were individually normalized to have zero mean and unit variance across all wavenumbers.

GOLD NANOROD PREPARATION FOR SERS MEASUREMENT

Hexadecyl(trimethyl)ammonium bromide (CTAB) and sodium oleate (NAOL) coated gold nanorods were synthesized following previously described protocol (*31*). The nanorods were cleaned by centrifuging 1 mL aliquots once at (9000 rpm, 20 min) and were concentrated down to 50 µL volumes. Nanorods were mixed with bacteria in 1:1 volumetric ratio to enable uniform coating as described in our earlier work (*8*). Absorption spectra were recorded using a Cary 5000 UV-vis-NIR spectrometer. Scanning electron microscopy images were taken using FEI Magellan 400 XHR Scanning Electron Microscope and the Zeiss Sigma Scanning Electron Microscope (SEM). Transmission electron microscopy images were taken using FEI Tecnai G2 F20 X-TWIN Transmission Electron Microscope (TEM).

CNN ARCHITECTURE AND CLASSIFICATION

Our CNN model is adapted from a ResNet (*19*) architecture, consisting of an initial convolutional layer with 64 filters, 7 residual layers, and one fully connected layer. Each residual layer consists of two residual blocks and each convolutional layer has 32 hidden nodes. We used a minibatch size of 32. To assess the classification performance, we used 10-fold cross validation, which splits the dataset into 10 equal stratified sets (Scikit-learn StratifiedKFold). For each fold i, we leave set i as the test set and train model i using all remaining data. Model i is then used to classify data in set i (test set). We repeat this process for all 10 folds, which produces unbiased classifications for all the data, and these are compared against the ground-truth to generate confusion matrices and the overall accuracy. All t-SNE projections were plotted using Scikit-learn manifold t-SNE with a perplexity = 15.

To determine the most important wavenumbers or spectral bands affecting classification performance, we used a probing algorithm that perturbs the input data before re-running the

classification through the trained models. Using the test fold from our 10-fold cross validation, we iterate through the wavenumbers and at each iteration, perturb the spectrum by modulating the amplitude of the spectral intensity with a Voigt distribution centered at the probing wavenumber. After each perturbation, we recalculate the classification accuracy, compare the updated results with our baseline classification accuracy, and determine the importance for each wavenumber - the greater the decrease in accuracy due to a given perturbation, the more important the wavenumber. Each wavenumber of each spectrum in the test fold is perturbed 5 times by randomly sampling spectral intensity values at that wavenumber in order to create random, but spectrally sensible instances. Finally, all results are averaged to determine our final feature importance.

DATA COLLECTION ON OCTOPI-RAMAN

Raman spectra were collected from 350 cm^{-1} to 1606 cm^{-1} using a 20x, 0.75 NA objective with spot size of 5.5 μm (Gaussian diameter; FWHM of 3.2 μm) , laser power of 2.53 mW , and 0.3 second acquisition time per spectrum.

SPUTUM SAMPLES

To obtain processed sputum for Raman interrogation, sputum samples were decontaminated and concentrated per standard laboratory procedure (*32*) as follows: the sputum samples were treated with N-acetyl-l-cysteine–sodium hydroxide (NALC-NaOH) to break up the mucus and treated with NaOH to kill the oral flora. The pH was then neutralized with phosphate buffer and samples were concentrated with centrifugation. Only sputa with a negative acid-fast bacilli microscopy result (mycobacteria-negative) and from patients at low risk for TB were used for BCG spiking.

Figs. S1 to S7:

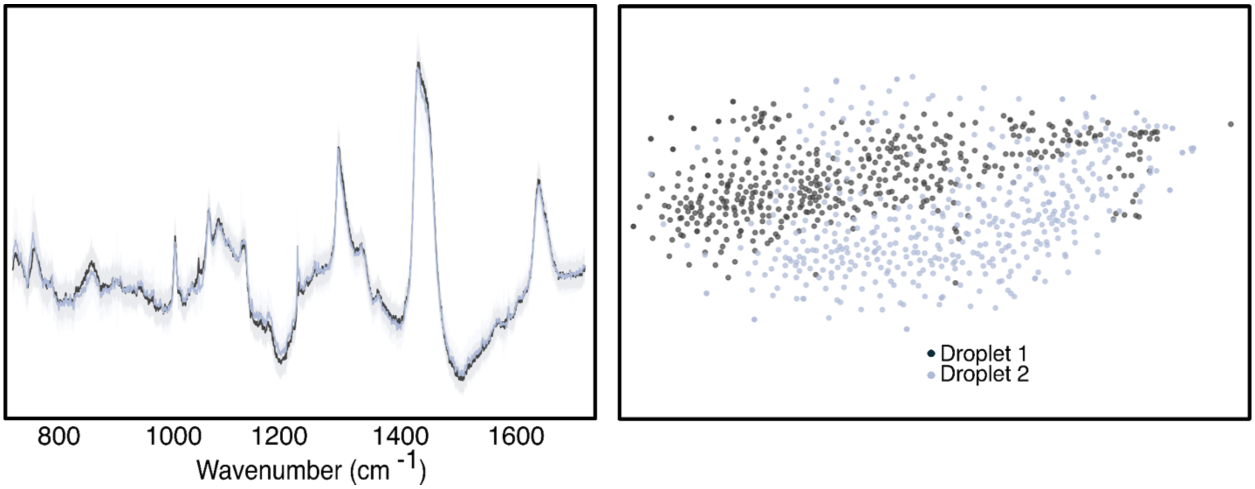

fig. S1. Droplet repeatability study. 450 Raman spectra of wildtype BCG collected on two separate droplets, showing spectral overlap and T-SNE overlap.

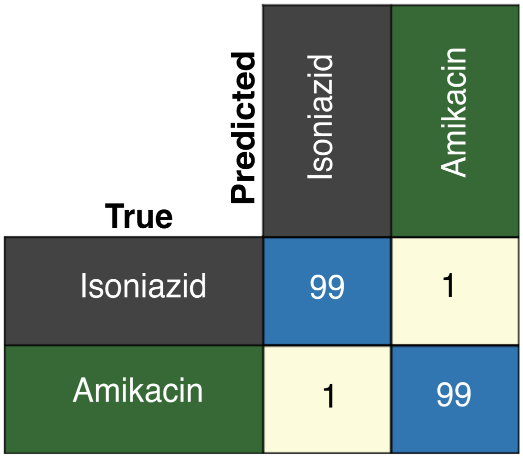

fig. S2. Confusion matrix classifying the isoniazid and amikacin resistant mutants when grouped according to their antibiotic class, showing ~99% classification accuracy (529 spectra per strain; 2116 spectra for the Isoniazid grouping and 1587 spectra for the Amikacin grouping)

True \ Predicted	Wildtype	Amikacin	Rifampicin	Isoniazid	Moxifloxacin
Wildtype	95			5	
Amikacin		97	1		2
Rifampicin	1	1	95	1	2
Isoniazid	5			94	1
Moxifloxacin		3	2		95

True \ Predicted	Wildtype	Amikacin	Rifampicin	Isoniazid	Moxifloxacin
Wildtype	96			4	
Amikacin		98			2
Rifampicin	1	1	95	1	2
Isoniazid	5			95	
Moxifloxacin		4	2	1	93

fig. S3. Confusion matrices from two replicate studies on the Horiba Labram scientific grade Raman microscope showing an average of ~95% accuracy across the 5 BCG strains (~450 and 529 spectra per strain collected, respectively).

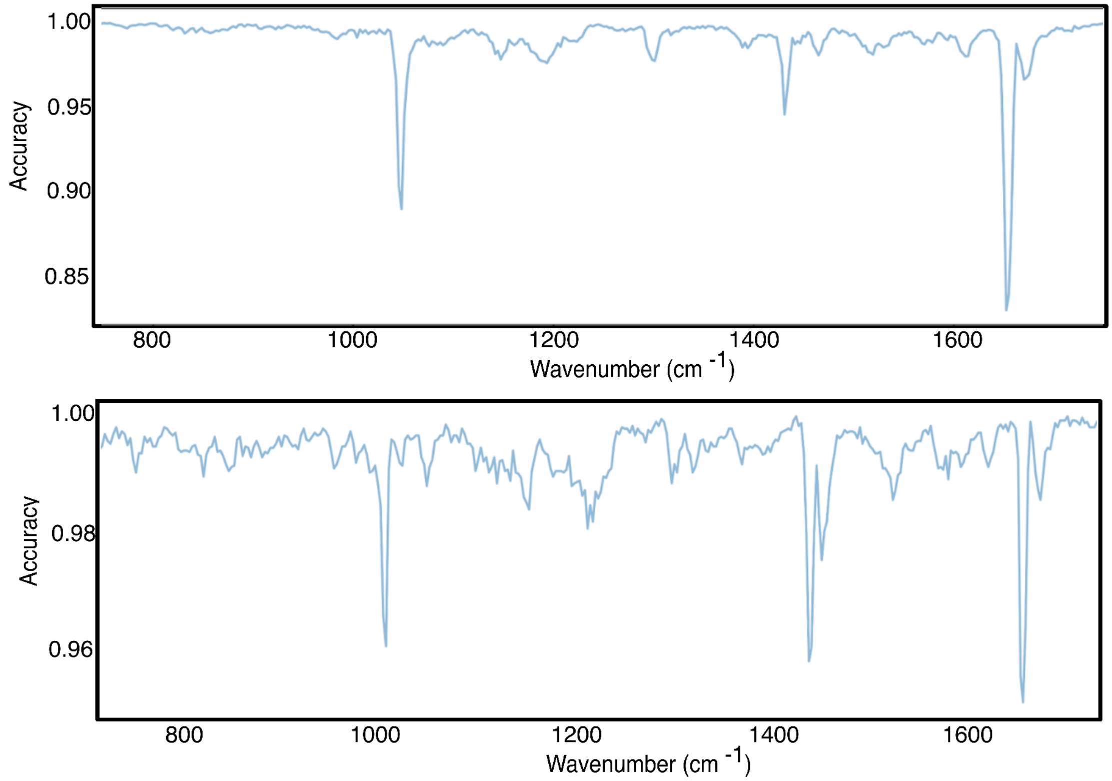

fig. S4. Feature selection from two replicate studies on the Labram.

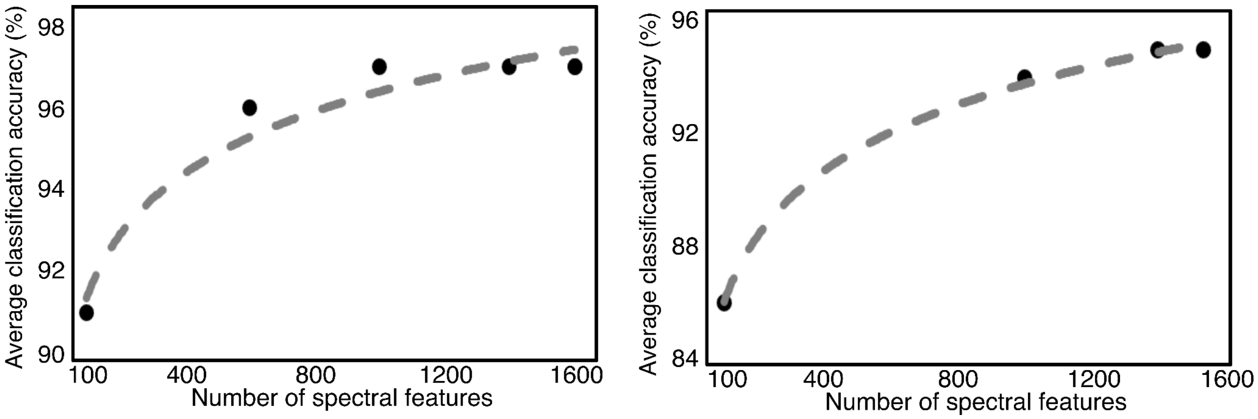

fig. S5. Average classification accuracy vs number of spectral features for two replicate Labram studies.

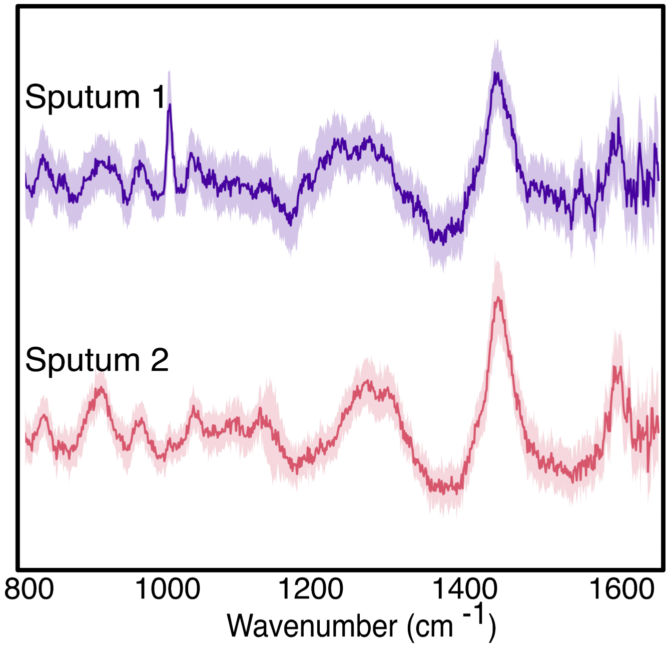

fig. S6. Average Raman spectra of the two sputum samples used in the Octopi study.

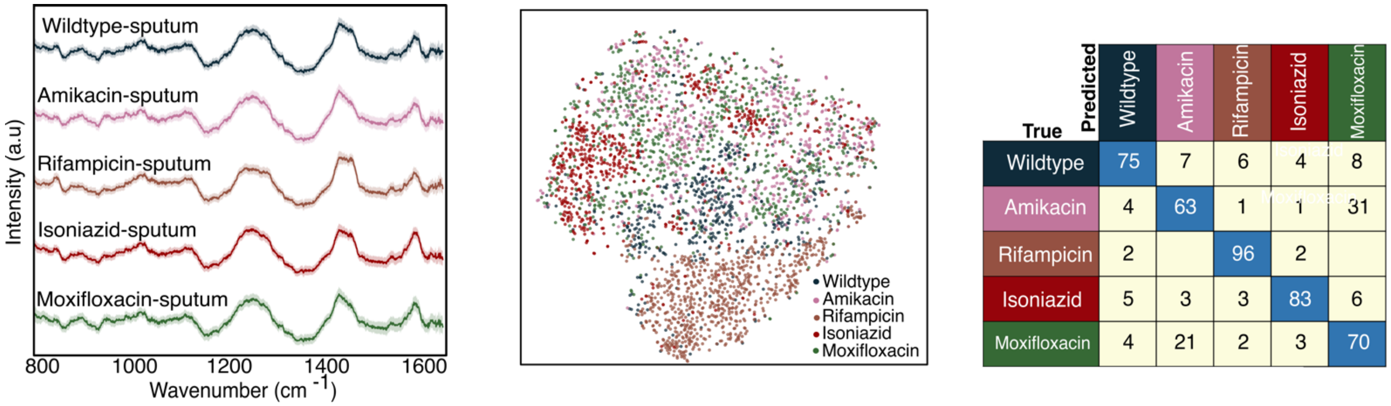

fig. S7. Mean Raman spectra of 5 BCG strains spiked in other sputum sample collected on Octopi-Raman with t-SNE showing some clustering by antibiotic class, and the confusion matrix demonstrating ~ 77% average classification accuracy.

Table S1:

MIC$_{90}$ ($\mu g/mL$)					
Antibiotic	Wildtype BCG	Amikacin-resistant BCG	Isoniazid-resistant BCG	Moxifloxacin-resistant BCG	Rifampicin-resistant BCG
Amikacin	0.0625	$>$ 4.0	0.0625	0.0625	0.0625
Isoniazid	0.5	0.5	$<$1	0.5	0.5
Moxifloxacin	0.016	0.016	0.016	1	0.016
Rifampicin	$<$0.008	$<$0.008	$<$0.008	$<$0.008	$<$4.0

table S1: Minimum inhibitory concentrations of the five BCG strains studied. This was obtained using standard serial two-fold dilution method.

References (*35, 36*):

35. X. Ye, C. Zheng, J. Chen, Y. Gao, C. B. Murray, Using binary surfactant mixtures to simultaneously improve the dimensional tunability and monodispersity in the seeded growth of gold nanorods. *Nano Lett.* **13**, 765–771 (2013).

36. K. C. Carroll, M. A. Pfaller, M. L. Landry, A. J. McAdam, R. Patel, S. S. Richter, D. W. Warnock, *Manual of Clinical Microbiology, 2 Volume Set* (Wiley, 2019).
